# Genetic Mutations and Clinical Severity in Hemoglobin E/Beta-Thalassemia Patients in Bangladesh

**DOI:** 10.7759/cureus.111264

**Published:** 2026-06-21

**Authors:** Nishat Mahzabin, Ismat Ara Islam, Mily Dey, Md Raiq Raihan Chowdhury, Md Kamrul Hasan Sajib, Md Abdul Aziz, Tawfika Rahman Jishan, Amin Lutful Kabir

**Affiliations:** 1 Department of Hematology, National Institute of Cancer Research and Hospital, Dhaka, BGD; 2 Department of Hematology, Sir Salimullah Medical College and Mitford Hospital, Dhaka, BGD; 3 Department of Hematology, Bangladesh Medical University, Dhaka, BGD; 4 Department of Medicine, Mugda Medical College and Hospital, Dhaka, BGD; 5 Epidemiology and Public Health, Pi Research and Development Center, Dhaka, BGD

**Keywords:** bangladesh, gene sequencing, genetic mutation characteristics, hb e/beta thalassemia, phenotype

## Abstract

Background and objective

The phenotypically diverse presentation of hemoglobin (Hb) E/β-thalassemia is often attributed to coinheritance of β-globin (HBB) gene mutations. This study aimed to describe the genetic mutations and clinical severity of HbE/β-thalassemia patients from low-resource settings.

Methods

A total of 32 HbE/β-thalassemia patients were included in this cross-sectional study. Cases were confirmed by capillary Hb electrophoresis or high-performance liquid chromatography and were further analyzed alongside clinical information and ancestral data. The data collection period spanned from May 2019 to July 2020. Gene sequencing was performed using the Sanger sequencing method for mutational analysis, and Mahidol scoring was applied to assess clinical severity.

Results

The median age of the patients was 20 years (interquartile range (IQR): 17-22.5). Phenotypically, mild, moderate, and severe disease were observed in 12 (37.5%), 14 (43.8%), and six (18.8%) patients, respectively. Overall, 13 heterozygous mutations were identified in the HBB gene. Among these, IVS-1-5 (G>C) was the most common mutation (n = 17, 53.1%), and codon 30 (G>C) (n = 4, 12.5%) was the second most common mutation. The IVS-1-5 (G>C) mutation was significantly more frequent among patients with severe disease (p<0.001).

Conclusions

This study is one of the few to characterize clinical severity and genetic mutations in HbE/β-thalassemia patients in Bangladesh. The information on clinical severity patterns could be useful for determining service priorities and ensuring the proper allocation of limited resources.

## Introduction

Thalassemia is one of the most common hemoglobin (Hb) disorders worldwide. Each year, over 332,000 conceptions are affected, with 56,000 developing clinically significant hemoglobinopathy [1]. Normal human Hb consists of four globin chains forming a tetramer. In this autosomal recessive disease, one or more globin chains of the Hb tetramer are reduced or absent, resulting in impaired Hb production [2]. In healthy adults, the majority of Hb (HbA) is produced by one pair each of α and β chains. Based on the globin chain involved, thalassemia is mainly classified as alpha (α) or beta (β) thalassemia. However, several other less common forms of thalassemia also exist. Hb variants may occur due to structural alterations in one of the globin chains [3]. The beta form of the disease is the most prevalent, affecting approximately 1.5% of the global population [4]. In contrast, HbE variants are the most common Hb variant worldwide [3]. South Asia is a hotspot for both β-thalassemia and the HbE variant, with an estimated 30 million carriers and one million affected (homozygous) mutants [4]. In Bangladesh, 11.89% of people carry β-globin gene mutations, among whom 8.68% have HbE traits, and 2.24% have beta-thalassemia traits [5].

HbE and β-thalassemia alleles are frequently co-inherited in the South Asian population due to the high prevalence of both mutations in this region. Although HbE itself produces a mild form of anemia, its interaction with different β-thalassemia mutations can result in a wide range of clinical manifestations [6]. The compound heterozygote HbE/β-thalassemia is commonly observed in India, Bangladesh, and throughout Southeast Asian countries [7]. HbE/β-thalassemia exhibits notable phenotypic variability, ranging from mild to severe disease [7]. Many genetic and environmental factors have been implicated in the clinical diversity of this condition. These include the type of β-thalassemia mutation coinherited with HbE, the coinheritance of hemoglobin F (HbF) modifier mutations, age-related adaptive changes, and the presence of malaria, among others [7,8].

Despite the high prevalence of HbE/β-thalassemia in Bangladesh [5], very few studies have documented the clinical severity and molecular characteristics of the disease in the country. Although several studies have attempted to describe the clinical presentation and mutational patterns of β-thalassemia [9-11], none have characterized the severity of the disease. One study applied the Mahidol scoring system to classify β-thalassemia intermedia patients [12]. In this study, we aimed to explore the clinical patterns and genetic mutations of HbE/β-thalassemia patients admitted to a tertiary care center in Bangladesh.

This article was previously posted to the Research Square preprint server on September 29, 2021.

## Materials and methods

Study setting and population

This cross-sectional study was conducted in the Department of Hematology, Bangladesh Medical University (BMU), Dhaka, Bangladesh, between May 2019 and July 2020. Diagnosed patients with HbE/β-thalassemia (confirmed by capillary Hb electrophoresis or high-performance liquid chromatography) who attended the hematology outpatient department and consented to genetic profiling were consecutively selected for inclusion. Patients with alloimmune reactions or concomitant chronic diseases (e.g., SLE, RA, CLD) that might interfere with the severity of thalassemia were excluded. A total of 32 HbE/β-thalassemia patients were included during the data collection period.

Data collection

Data were collected by two trained physicians using a semi-structured questionnaire consisting of two parts: (a) demographic and clinical features, and (b) laboratory investigations, including genetic analysis.

Clinical features and severity scoring

Several scoring systems have been developed to classify the clinicopathological heterogeneity of thalassemia [13]. We used the Mahidol scoring system, developed by Srichipai et al. at Mahidol University, Thailand [14], which is one of the earliest clinical severity scoring systems for thalassemia patients. It is a comprehensive scoring tool that considers the steady-state Hb level, age at initiation of transfusions, number of transfusions required, spleen size, age at first presentation, and growth retardation during the calculation of the score. A score of 0 to <4 is defined as mild, 4 to 7 as moderate, and >7 to 10 as severe disease. The details of the scoring system can be found in the original work by Srichipi et al. [14]. Transfusion dependency was defined as a regular requirement for transfusion to maintain survival [15,16]. Growth was assessed using the CDC growth charts [17]. Growth retardation was defined as weight-for-age and/or height-for-age below the third percentile. Steady-state Hb was calculated as the average Hb level from previous records prior to blood transfusion [14].

Laboratory investigations 

Patients were investigated for hematological profiles, capillary Hb electrophoresis, and gene sequencing for DNA analysis and mutation detection using Sanger sequencing. A complete blood count (CBC) was carried out using an automated hematology analyzer (Pentra ABX-120 DX, HORIBA Medical, Montpellier, France), hemoglobin electrophoresis was performed using a Sebia capillary electrophoresis system (Sebia S.A., Lisses, France), and gene sequencing was conducted using a genetic analyzer (3500 Genetic Analyzer, CE, IVD, Applied Biosystems, Waltham, MA).

Statistical analysis

All data were checked and entered into IBM SPSS Statistics version 26 (IBM Corp., Armonk, NY) for analysis. Categorical variables were expressed as frequencies (percentages), and continuous variables were expressed as mean ± standard deviation (SD) or median (interquartile range (IQR)), as appropriate. Bivariate analysis was conducted using Fisher’s exact test for comparison between two categorical variables and the Kruskal-Wallis test to compare median Hb values across broad mutation categories. A p-value of <0.05 was considered statistically significant.

Ethical measures

Ethical clearance for the study was obtained from the Ethical Review Committee of Bangabandhu Sheikh Mujib Medical University (BSMMU) (No. BSMMU/2019/7579). All the study procedures were conducted in line with the ethical principles laid out by the Declaration of Helsinki. Informed written consent was obtained from the patients or their guardians, if the patient was a minor, before inclusion.

## Results

**Figure 1:**
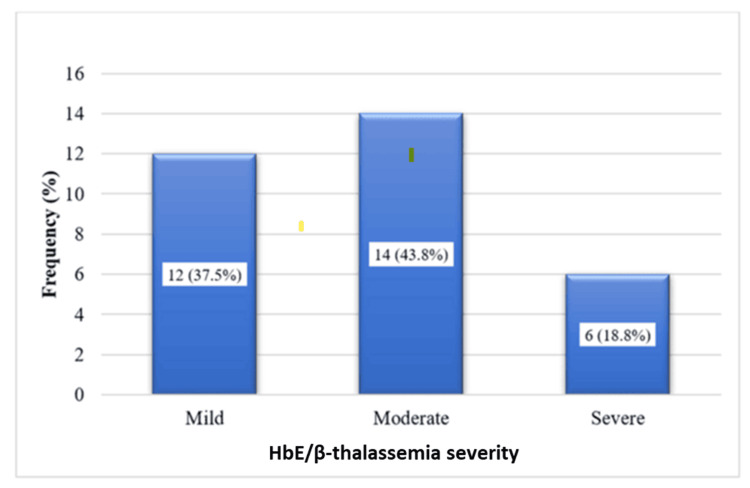
Distribution of patients by severity of HbE/β-thalassemia as assessed using the Mahidol scoring system (n = 32) HbE: hemoglobin E

A total of 32 confirmed cases of HbE/β-thalassemia were included in the study. According to the Mahidol scoring system, 37.5% (n = 12) of patients were classified as phenotypically mild, 43.8% (n = 14) as moderate, and 18.8% (n = 6) as severe (Figure 1).

The median age of the patients was 20 years (IQR: 17 - 22.5). Confirmation of the diagnosis and first blood transfusion occurred at a median age of 9.5 (IQR: 5 - 19.5) and 11 (IQR: 5 - 16) years, respectively. Patients diagnosed at a younger age initiated their first blood transfusion earlier than those diagnosed later. Severe disease, as assessed by the Mahidol criteria, was associated with a higher frequency of blood transfusions. Overall, 59.4% of patients were male, with a higher proportion in the moderate severity group. Only four patients (12.5%) had parents with consanguinity. However, 34.4% of patients were transfusion-dependent, receiving a median of 9.5 (IQR: 2.3 - 43.5) units of blood. The overall transfusion requirement increased with disease severity. Fifteen patients had completed growth. The average steady-state Hb was 7.6 ± 1.4 g/dl (Table 1).

**Table 1:** Demographic and clinical features by disease severity (n = 32) ^*^Transfusion dependency is defined as a regular requirement of blood transfusion for survival. ^**^Growth retardation is defined as weight for age and/or height for age below the third percentile. ^***^Steady-state hemoglobin was calculated as the average hemoglobin level from previous records before blood transfusion HbE: hemoglobin E; IQR: interquartile range; SD: standard deviation

Variable	Total	Severity of HbE/β-thalassemia		
		Mild (n = 12)	Moderate (n = 14)	Severe (n = 6)
Age, years, median (IQR)	20 (17 – 22.5)	20 (14.5 – 23)	19.5 (17 – 21.3)	22 (17.3 – 33.3)
Age at diagnosis, median (IQR)	9.5 (5 – 19.5)	15 (12 – 22.8)	6 (5.8 – 19.3)	3 (2 – 5.3)
Age at which transfusion started, median (IQR)	11 (5 – 16)	15 (13 – 19.5)	8.5 (5.8 – 19.0)	3.5 (2.8 – 8)
Weight, kg, mean ± SD	45.9 ± 14.1	53.3 ± 15.3	42.1 ± 11.3	40 ± 13.4
Height, cm, mean ± SD	146.8 ± 24.5	153.7 ± 23.6	143.6 ± 26.1	140.3 ± 22.9
Sex, n (%)				
Male	19 (59.4)	6 (50.0)	10 (71.4)	3 (50.0)
Female	13 (40.6)	6 (50.0)	4 (28.6)	3 (50.0)
Blood group, n (%)				
A positive	6 (18.8)	5 (41.7)	1 (7.1)	0
B positive	12 (37.5)	1 (8.3)	8 (57.1)	3 (50.0)
O positive	7 (21.9)	1 (8.3)	3 (21.4)	3 (50.0)
AB positive	7 (21.9)	5 (41.7)	2 (14.3)	0
Consanguineous parents, n (%)				
Present	4 (12.5)	2 (16.7)	1 (7.1)	1 (16.7)
Absent	28 (87.5)	10 (83.3)	13 (92.9)	5 (83.3)
Transfusion dependency^*^, n (%)				
Transfusion-dependent	11 (34.4)	1 (8.3)	5 (35.7)	5 (83.3)
Non-transfusion-dependent	21 (65.6)	11 (91.7)	9 (64.3)	1 (16.7)
Number of transfusions required last year, median (IQR)	3 (1 – 9)	0.5 (0 – 1.8)	4 (2.5 – 9.0)	14 (9 – 18.5)
Number of transfusions required in lifetime, median (IQR)	9.5 (2.3 – 43.5)	1.5 (0 – 6.5)	23.5 (5.0 – 40.5)	175 (54.3 – 317.5)
Growth Retardation^**^, n (%)				
Present	17 (53.1)	1 (8.3)	10 (71.4)	6 (100)
Absent	15 (46.9)	11 (91.7)	4 (28.6)	0
Hepatomegaly, n (%)				
Present	17 (53.1)	2 (16.7)	9 (64.3)	6 (100)
Absent	15 (46.9)	10 (83.3)	5 (35.7)	0
Splenomegaly, n (%)				
Present	28 (93.3)	11 (91.7)	12 (92.3)	5 (100)
Absent	2 (6.7)	1 (8.3)	1 (7.7)	0
Splenectomy, n (%)				
Done	2 (6.3)	0	1 (7.1)	1 (16.7)
Not done	30 (93.8)	12 (100)	13 (92.9)	5 (83.3)
Steady-state hemoglobin, g/dl^***^, mean ± SD	7.6 ± 1.4	8.8 ± 1.0	7.2 ± 1.1	6.2 ± 0.8

Table 2 shows the clinical presentation of patients according to the Mahidol scoring system. The majority of patients had a steady-state hemoglobin level >7.5 g/dL (53.1%), an age at first blood transfusion >10 years (56.3%), an occasional requirement for blood transfusion (53.1%), a spleen size >10 cm (37.5%), and an age at thalassemia presentation >10 years or between 2-10 years (46.9% each). Growth and development were below the third percentile in 50.0% of patients.

**Table 2:** Clinical presentation of patients according to the Mahidol scoring system ^*^Percentile of growth development was assessed based on weight and height measurements plotted on a CDC standard growth chart CDC: Centers for Disease Control and Prevention

Mahidol criteria		Score	Total	Mild (n = 12)	Moderate (n = 14)	Severe (n = 6)
			N (%)	N (%)	N (%)	N (%)
Steady-state hemoglobin, g/dl						
>7		0	17 (53.1)	11 (91.7)	6 (42.9)	0
6 – 7		1	14 (43.8)	1 (8.3)	8 (57.1)	5 (83.3)
<6		2	1 (3.1)	0 (0.0)	0 (0.0)	1 (16.7)
Age at receiving first blood transfusion, years						
>10		0	18 (56.3)	11 (91.7)	6 (42.9)	1 (16.7)
4 – 10		1	9 (28.1)	0 (0.0)	7 (50.0)	2 (33.3)
<4		2	5 (15.6)	1 (8.3)	1 (7.1)	3 (50.0)
Requirement for blood transfusion						
None/rare		0	4 (12.5)	4 (33.3)	0 (0.0)	0 (0.0)
Occasionally		1	17 (53.1)	8 (66.7)	8 (57.1)	1 (16.7)
Regularly		2	11 (34.4)	0 (0.0)	6 (42.9)	5 (83.3)
Size of spleen, cm						
<4		0	9 (28.1)	5 (41.7)	3 (21.4)	1 (16.7)
4 – 10		1	11 (34.4)	6 (50.0)	4 (28.6)	1 (16.7)
>10		2	12 (37.5)	1 (8.3)	7 (50.0)	4 (66.7)
Age at thalassemia presentation, years						
>10	0		15 (46.9)	10 (83.3)	5 (35.7)	0 (0.0)
2 – 10	0.5		15 (46.9)	1 (8.3)	8 (57.1)	6 (100.0)
<2	1		2 (6.3)	1 (8.3)	1 (7.1)	0 (0.0)
Growth and development^*^						
>25th percentile	0		10 (31.3)	9 (75.0)	1 (7.1)	0 (0.0)
3rd to 25th percentile	0.5		6 (18.8)	2 (16.7)	4 (28.6)	0 (0.0)
<3^rd^ percentile	1		16 (50.0)	1 (8.3)	9 (64.3)	6 (100.0)

**Figure 2:**
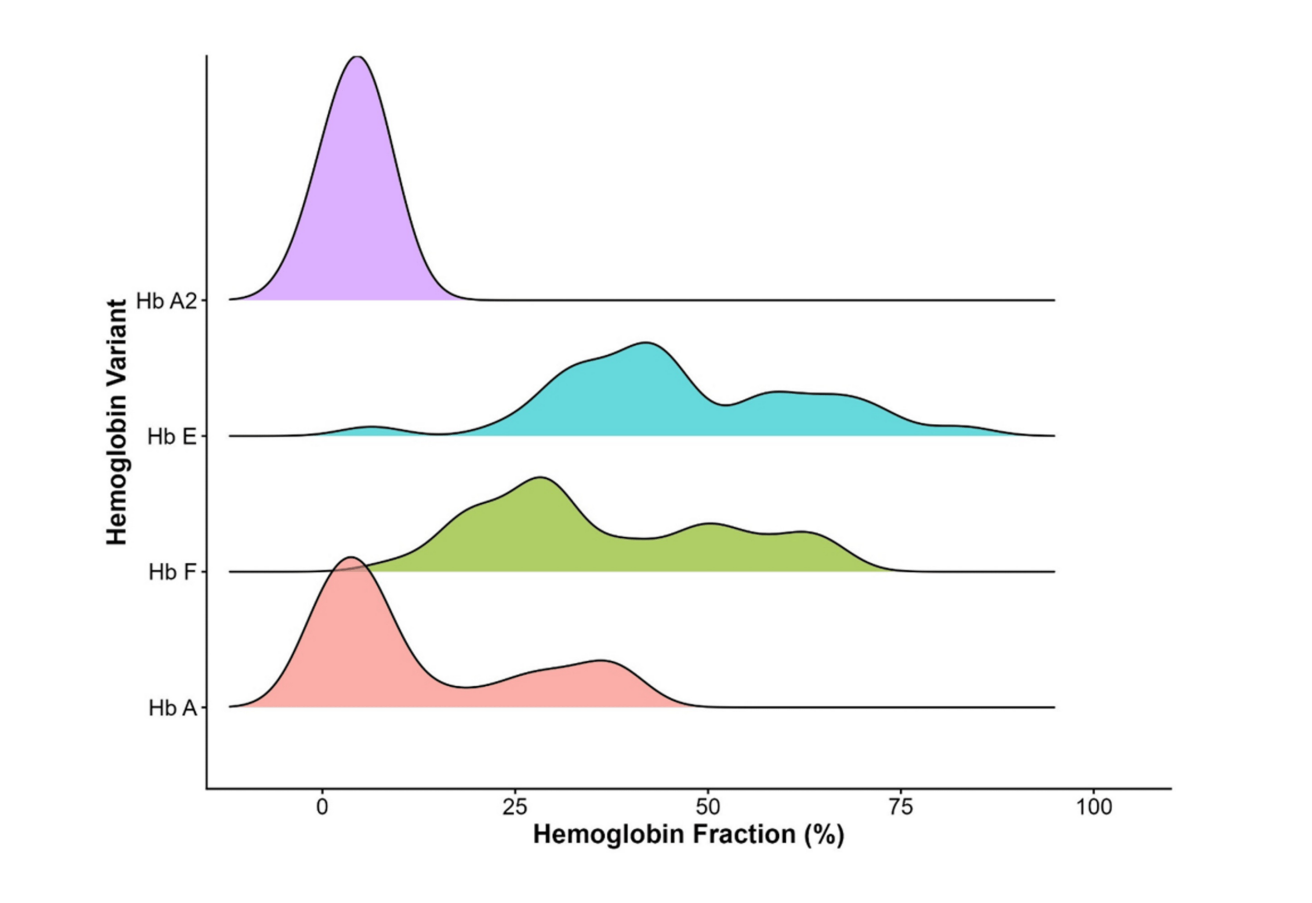
Ridgeline plots showing the distribution of Hb types among all patients (n = 32) Hb: hemoglobin

**Figure 3:**
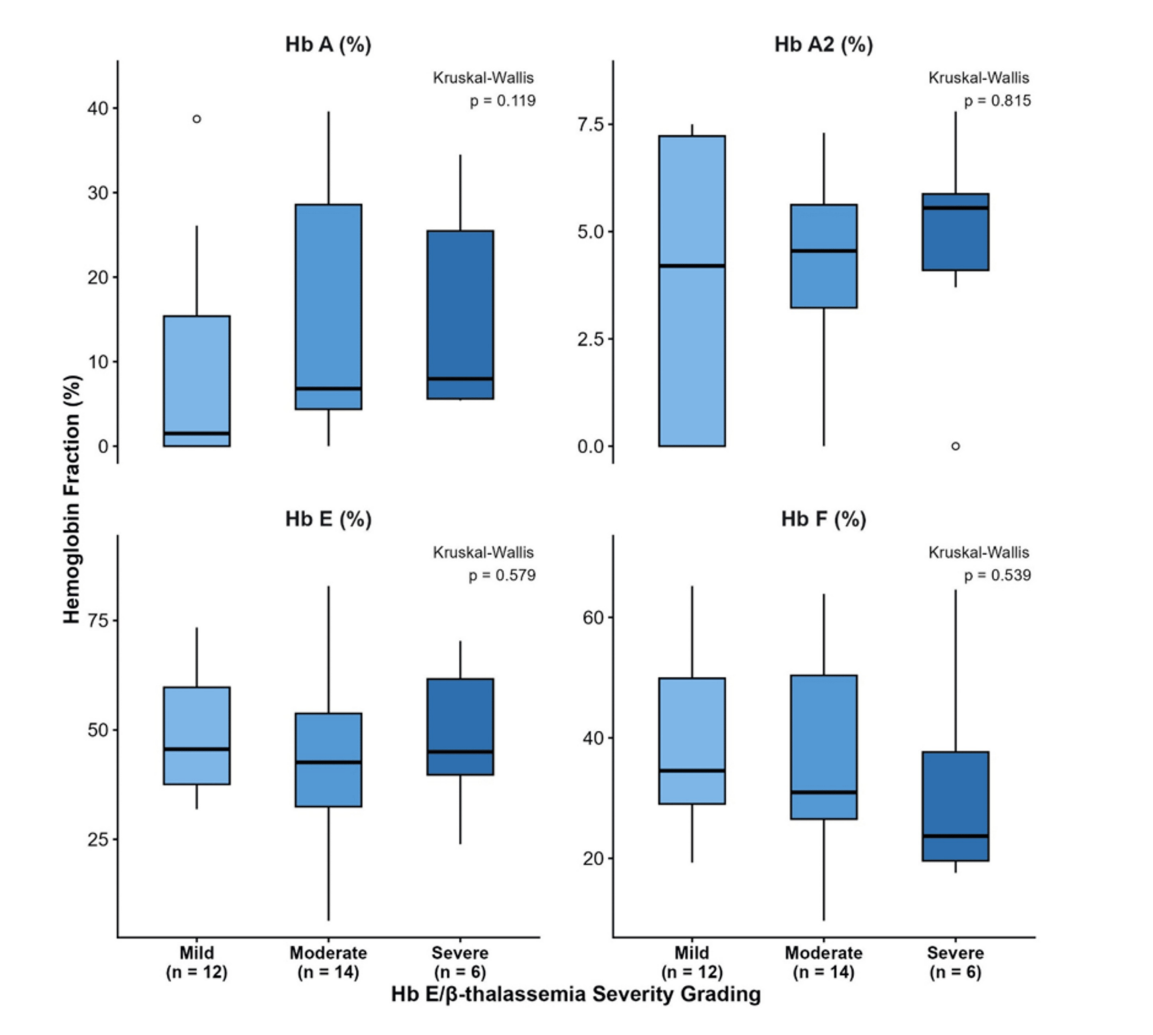
Boxplots showing distribution of hemoglobin fractions by severity in HbE/β-thalassemia patients (n = 32) HbE: hemoglobin E

Figures 2-3 show the distribution of four types of hemoglobin (HbA, HbF, HbE, and HbA2) in general and across severity grades of thalassemia, respectively. HbF and HbE exhibited a relatively wider distribution compared with HbA and HbA2 across patients. HbA and HbA2 were expressed at levels as high as 50% and 25%, respectively. However, HbE expression was the most dominant among all hemoglobin fractions (Figure 2). The median hemoglobin fractions showed a statistically similar distribution across the severity of thalassemia (Figure 3).

**Figure 4:**
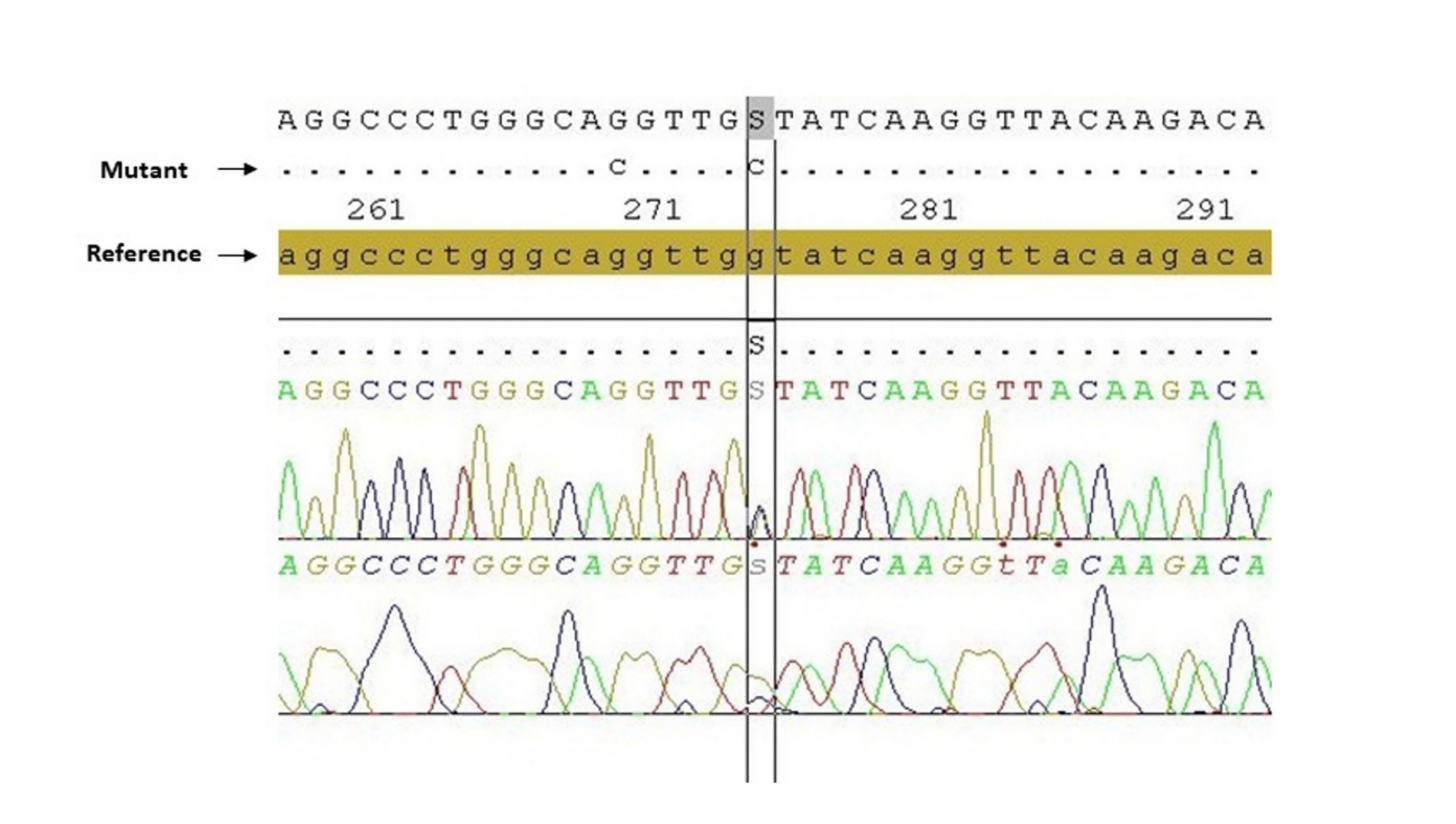
Chromatogram of a patient with HbE/β-thallasemia determined by the automated Sanger sequencing machine HbE: hemoglobin E

In total, 13 different heterozygous beta-globin chain mutations were identified. Point mutation IVS-1-5 (G>C) (n = 17, 53.1%) and codon 30 (G>C) (n = 4, 12.5%) mutations were the most common. The genetic mutations of all patients are summarized in Table 3. Figure 4 shows an example chromatogram from a patient with an IVS-1-5 (G>C) mutation.

**Table 3:** Observed mutations (total 13) in HbE/β-thalassemia participants in the study HbE: hemoglobin E; IVS: Intervening sequence; bp: base pair

Allelic status	Mutation	Type of mutation	N (%)
Heterozygous	IVS-1-5 (G>C)	Point mutation	17 (53.1)
Heterozygous	Codon 30 (G>C)	Point mutation	4 (12.5
Heterozygous	Codon 15 (G>A)	Point mutation	1 (3.1)
Heterozygous	Codon -90 (C>T)	Point mutation	1 (3.1)
Heterozygous	Codon 110 (T>C)	Point mutation	1 (3.1)
Heterozygous	IVS1-130 (G>C)	Point mutation	1 (3.1)
Heterozygous	IVS-2-1 (G>A)	Point mutation	1 (3.1)
Heterozygous	619-bp deletions	Deletion	1 (3.1)
Heterozygous	Codon 15 (-G)	Point deletion	1 (3.1)
Heterozygous	Codon 15 (-T)	Point deletion	1 (3.1)
Heterozygous	Codon 16 (-C)	Point deletion	1 (3.1)
Heterozygous	Codon 77/78 (+C)	Point insertion	1 (3.1)
Heterozygous	Codon 41/42 (-TTCT)	Deletion	1 (3.1)

Of the 12 patients in the mild group, three were characterized by heterozygous codon 30 (G>C), while the remaining patients carried heterozygous codon -90 (C>T), heterozygous codon 110 (T>C), heterozygous IVS-1-130 (G>C), heterozygous 619-bp deletion, heterozygous codon 15 (-G), heterozygous codon 15 (-T), heterozygous codon 16 (-C), heterozygous codon 77/78 (+C), and heterozygous codon 41/42 (-TTCT). Of the 14 patients in the moderate severity group, most had heterozygous IVS-1-5 (G>C) (n = 11), one had heterozygous codon 30 (G>C), one had heterozygous codon 15 (G>A), and one had heterozygous IVS-2-1 (G>A). Among the six patients in the severe group, all were characterized exclusively by heterozygous IVS-1-5 (G>C), with no other mutations detected. The heterozygous IVS-1-5 (G>C) mutation was significantly associated with disease severity (p<0.001) (Table 4).

**Table 4:** Association of HBB gene mutations with clinical severity of HbE/β-thalassemia P-values were determined using Fisher’s exact test HbE: hemoglobin E

Mutations	Clinical severity, n (%)			P-value
	Mild (n = 12)	Moderate (n = 14)	Severe (n = 6)	
Heterozygous for IVS-1-5 (G>C)	0 (00.0)	11 (64.7)	6 (35.3)	<0.001
Heterozygous for codon 30 (G>C)	3 (75.0)	1 (25.0)	0 (00.0)	0.375
Heterozygous for codon 15 (G>A)	0 (00.0)	1 (100.0)	0 (00.0)	1.000
Heterozygous for codon -90 (C>T)	1 (100.0)	0 (00.0)	0 (00.0)	0.563
Heterozygous for codon 110 (T>C)	1 (100.0)	0 (00.0)	0 (00.0)	0.563
Heterozygous for IVS-1-130 (G>C)	1 (100.0)	0 (00.0)	0 (00.0)	1.000
Heterozygous for IVS-2-1 (G>A)	0 (00.0)	1 (100.0)	0 (00.0)	0.563
Heterozygous for 619-bp deletions	1 (100.0)	0 (00.0)	0 (00.0)	0.563
Heterozygous for codon 15 (-G)	1 (100.0)	0 (00.0)	0 (00.0)	0.563
Heterozygous for codon 15 (-T)	1 (100.0)	0 (00.0)	0 (00.0)	0.563
Heterozygous for codon 16 (-C)	1 (100.0)	0 (00.0)	0 (00.0)	0.563
Heterozygous for codon 77/78 (+C)	1 (100.0)	0 (00.0)	0 (00.0)	0.563
Heterozygous for Codon 41/42(-TTCT)	1 (100.0)	0 (00.0)	0 (00.0)	0.563

Table 5 shows the percentage of different types of hemoglobin found in electrophoresis in relation to major mutation types. The median HbF proportion of IVS-1-5 (G>C), Codon 30 (G>C), and other mutations were 30.5%, 56.55%, and 29.6%, respectively. However, the difference was not statistically significant. Similarly, the proportion of HbA, HbE, and HbA2 showed statistically non-different distribution across mutation categories.

**Table 5:** Association of HBB gene mutations with types of Hb found among patients P-values were determined using the Kruskal-Wallis test Hb: hemoglobin

Hemoglobin type	Mutations, median (min-max)			H-statistic	P-value
	Heterozygous for IVS-1-5 (G>C) (n = 17)	Heterozygous for Codon 30 (G>C) (n = 4)	Other heterozygous mutations (n = 11)		
HbA (%)	6.1 (0 – 39.6)	1.5 (0 – 29.7)	7.0 (0 – 38.7)	2.327	0.312
HbF (%)	30.5 (9.6 – 64.6)	56.55 (38.9 – 63.9)	29.6 (19.3 – 65.2)	4.946	0.084
HbE (%)	42.6 (23.9 – 82.9)	40.4 (6.4 – 55.8)	46.9 (31.9 – 73.4)	1.879	0.391
HbA2 (%)	4.8 (0 – 7.8)	1.6 (0 – 5.3)	5.9 (0- 7.5)	3.018	0.221

A detailed breakdown of clinical features (as used in the Mahidol scoring system) by HBB gene mutations shows that patients heterozygous for IVS-1-5 (G>C) mutation had a higher frequency of blood transfusion, enlarged spleen (>10 cm), and growth retardation (<3rd percentile). However, the steady-state hemoglobin of these patients remained mostly between 6 and 7.5 g/dl (see table in the Appendices).

## Discussion

HbE/β-thalassemia exhibits phenotypic heterogeneity, with presentations ranging from mild to severe disease [3]. Our study also reflected this variability in clinical features. We found that nearly two-thirds of patients had moderate to severe disease according to the Mahidol scoring system. This is consistent with the original study that developed the scale at Mahidol University, Thailand, which reported that more than two-thirds of patients had moderate to severe disease in a large sample of 950 patients [14]. In contrast, an Indonesian study found that nearly four-fifths of patients had moderate to severe disease using the same scale, which is higher than the proportion observed in our study.

Although no prior classification had been performed among HbE/β-thalassemia patients in Bangladesh, the Mahidol score was previously applied to classify β-thalassemia intermedia patients in the country. In that study, Mannan et al. reported that 35.3% of patients had moderate disease and 6% had severe disease. Various genetic interactions underlie intermediate forms of β-thalassemia, and this form appears to be phenotypically less severe than forms compounded by HbE variants [3]. Nonetheless, the reasons for such variability in presentations are not fully understood and remain an active area of research [18].

We noted a wide age range at which the diagnosis was made, and transfusions were started among the patients. An earlier age at presentation was usually associated with a higher severity score requiring a higher number of transfusions and a higher proportion of growth retardation and hepatosplenomegaly among HbE/β-thalassemia patients. Olivieri et al. [7] extensively studied the phenotypic variability of such a group of thalassemic patients, which endorses our findings. Also, male patients were more frequent in our study, congruent with previous estimates [13].

The IVS-1-5 (G>C) mutation was the most common mutation, found in more than 50% of participants in this study. Mutational analysis of β-thalassemic individuals by Ayub et al. [10] also reported that the splice site mutation IVS-1-5 (G>C) was the most common mutation in Bangladesh. Furthermore, this mutation is the most prevalent among thalassemic patients in South Asia [19]. Previous studies [7,18] have shown heterogeneity in the distribution of HBB gene mutations in relation to the phenotypic variability of HbE/β-thalassemia. However, we observed a higher frequency of the IVS-1-5 (G>C) mutation among patients with moderate to severe disease. Notably, all patients with severe phenotypes in our study carried this mutation.

We know that the IVS-1-5 (G>C) mutation produces β+-thalassemia alleles, leading to defective mRNA splicing and impaired globin chain production [20]. This, when compounded by the HbE mutation (codon 26 G>A), leads to impaired HbA production, increased HbA2 and HbE synthesis, and an overall reduction in hemoglobin levels [21]. It could be argued that the proportion of HbF in these patients may have been low due to the absence of positive variants in the Xmn1, BCL11A, HBS1L, or MYB genes [22,23]. However, the similar HbF levels observed in IVS-1-5 (G>C) mutants compared with other mutants in our study challenge this explanation. Additionally, the possibility that alloimmune reactions might have falsely increased disease severity in our patients is excluded because of the screening out of such patients before inclusion in this study. 

However, one might argue that the presence of IVS-1-5 (G>C) mutations across all severities of presentation in HbE/β-thalassemia patients, as described by Olivieri et al. [24], argues against a direct association. Their findings suggest that other secondary and tertiary modifiers, including genetic and environmental attenuating factors, may influence the phenotypic heterogeneity of the same mutation [8]. Furthermore, the classification schemes used for severity grading may have affected the distribution observed in Olivieri et al. [24]. As we could not characterize α-globin gene mutations among our participants, assessing the potential moderating effect of α-globin mutations on disease severity [7] was not possible in this study. Therefore, further studies with larger sample sizes are warranted to clarify any association.

Finally, one case among our participants warrants particular attention to highlight the lack of awareness in thalassemic patients in a low-resource setting. Interestingly, this patient had a Mahidol score classified as severe but received only occasional blood transfusions. This 20-year-old daughter of non-consanguineous parents presented with severe anemia, a massively enlarged spleen, and severe growth retardation (<3rd percentile). Her age at first presentation was six years, but she did not begin transfusions until age 17. Investigations revealed a low steady-state hemoglobin of 5 g/dL, a spleen size of 18 cm, HbA, HbF, and HbE levels of 10.33%, 19.32%, and 70.35%, respectively, and an IVS-1-5 (G>C) mutation. Her high levels of HbE may have partially protected her against severe anemia, as HbE retains some functionality [6]. However, the prolonged delay in initiating transfusions reflects a lack of awareness and follow-up, which likely contributed to her overt clinical presentation.

A general lack of awareness about thalassemia and inadequate access to dedicated treatment facilities due to geographic and/or poor socioeconomic conditions often lead to delayed presentation or follow-up at hospitals in resource-poor settings. As Hossain et al. [19] noted in a review of thalassemia in South Asian countries, health awareness is very poor among the general population, and many patients with thalassemia may die without ever knowing about their disease. Nonetheless, this case also illustrates the wide range of presentations in HbE/β-thalassemia patients and emphasizes the importance of early detection and regular follow-up.

The major limitation of our study is the small sample size and the characterization of patients from a single center. Another limitation is the inability to characterize α-globin gene mutations and other single-nucleotide polymorphisms (SNPs) in the patients. However, our study is one of the earliest attempts to explore an association between phenotypic severity and HBB gene mutations in HbE/β-thalassemia patients, and our findings should encourage further exploration of the relationship between genetic diversity and clinical severity in thalassemic patients in the country.

## Conclusions

HbE/β-thalassemia patients of this study mostly presented with moderate to severe disease, as determined by the Mahidol scoring system. The splice site mutation IVS-1-5 (G>C) was the most common and was frequently associated with severe presentations. As one of the South Asian countries with a high prevalence of HbE traits, the coexistence of β-thalassemia is expected in Bangladesh. By highlighting the variability in patient presentations, our findings emphasize the importance of screening for and diagnosing severe phenotypes, as well as investigating the association between genetic variation and clinical severity in large-scale, multicenter studies.

## Appendices

**Table 6:** Distribution of HBB mutations by clinical severity ^*^Growth retardation is defined as weight for age and/or height for age below the third percentile

	Steady-state hemoglobin (g/dl)			Age at receiving first blood transfusion (years)			Requirement for blood transfusion			Size of spleen (cm)			Age at thalassemia presentation (years)			Growth and development^*^		
HBB mutation	>7	6 – 7	<6	>10	4 – 10	<4	None/rare	Occasionally	Regularly	<4	4 – 10	>10	>10	2 – 10	<2	>25th percentile	3rd to 25th percentile	<3rd percentile
heterozygous for IVS1-5 (G>C)	5	11	1	4	9	4	0	8	9	4	3	10	3	13	1	1	3	13
heterozygous for codon 30 (G>C)	3	1	0	4	0	0	2	1	1	0	2	2	4	0	0	2	0	2
heterozygous for codon 15 (G>A)	1	0	0	1	0	0	0	1	0	0	1	0	0	1	0	0	0	1
heterozygous for -90 (C>T)	1	0	0	0	0	1	0	1	0	1	0	0	0	0	1	1	0	0
heterozygous for codon 110 (T>C)	1	0	0	1	0	0	0	1	0	0	1	0	1	0	0	1	0	0
heterozygous for IVS 1-130 (G>C)	0	1	0	1	0	0	0	0	1	0	1	0	1	0	0	0	1	0
heterozygous for IVS 2-1 (G>A)	1	0	0	1	0	0	0	1	0	1	0	0	0	1	0	1	0	0
heterozygous for 619 bp deletion	1	0	0	1	0	0	0	1	0	0	1	0	1	0	0	1	0	0
heterozygous for codon 15 (-G)	1	0	0	1	0	0	1	0	0	1	0	0	1	0	0	1	0	0
heterozygous for codon 15 (-T)	1	0	0	1	0	0	0	1	0	1	0	0	1	0	0	0	1	0
heterozygous for codon 16 (-C)	0	1	0	1	0	0	0	1	0	0	1	0	1	0	0	1	0	0
heterozygous for codon 77/78 (+C)	1	0	0	1	0	0	0	1	0	0	1	0	1	0	0	0	1	0
heterozygous for codon 41/42 (-TTCT)	1	0	0	1	0	0	1	0	0	1	0	0	1	0	0	1	0	0
